# Validity of scoring system for palliative care in oncology: CODETM – “Care of the dying evaluation”. Is it important in assessing the end-of-life process?

**DOI:** 10.1590/0102-67202025000046e1915

**Published:** 2026-01-09

**Authors:** Thayssa de Morais OLIVEIRA, Juliana Nalin PASSARINI, Dagny Faksvåg HAUGEN, Catriona Rachel MAYLAND, Luiz Roberto LOPES

**Affiliations:** 1 Universidade Estadual de Campinas, Department of Surgery, Faculty of Medical Sciences – Campinas (SP), Brazil.; 2 Universidade Estadual de Campinas, Department of Clinical Medicine, Faculty of Medical Sciences – Campinas (SP), Brazil.; 3 University of Bergen, Department of Clinical Medicine K1 – Bergen, Norway.; 4 University of Liverpool, Palliative Care Institute – Liverpool, United Kingdom.

**Keywords:** Neoplasms, Palliative Care at the End of Life, Questionnaire, Neoplasias, Cuidados Paliativos na Terminalidade da Vida, Questionário

## Abstract

**Background:**

Patients with advanced cancer experience a range of distressing symptoms. Palliative care (PC) emerges as an essential area to be implemented by health systems in the care of patients with irreversible diseases and beyond therapeutic possibilities.

**Aims:**

To compare the perception of caregivers of patients in palliative care offered by two public hospitals using the CODE^TM^ questionnaire; to determine the score obtained by the questionnaire and its usefulness in the evaluation of the palliative care offered.

**Methods:**

The post-death questionnaire “Care of the Dying Evaluation” (CODE^TM^) was applied to the family members who accompanied the patients in the last days, assessing the perception of the quality of care provided to the patient and the level of support to the family.

**Results:**

No statistical difference in demographics. Participants who received palliative care had higher scores in the score, as well as in the ward and ICU unit compared to the emergency unit. The predictive cut-off value for adequate palliative care practice was 97 points, corresponding to 78.6% of the score.

**Conclusions:**

There was no statistical difference between the caregivers’ perception of the care offered to patients between the two hospitals, being worse in the emergency unit. The cut-off value was 78.6% and was considered adequate and the CODE^TM^ questionnaire was a useful tool in the evaluation of palliative care offered by hospitals to patients and can be applied to propose improvements in palliative care. Therefore, there is a need for an instrument that can constantly classify and qualify the care provided to patients and their families in order to offer dignified, comprehensive and humanized care, as proposed by the CODE^TM^ questionnaire

## ARTICLE HIGHLIGHTS

- Patients with advanced cancer experience a range of distressing symptoms.
- Palliative care is emerging as a key area for healthcare systems to innovate in assisting patients with irreversible diseases beyond therapeutic possibilities.
- Is an evaluation of palliative care provided at the end of life to SUS patients by public health institutions necessary? The answer is yes.
- For this evaluation, the CODE™ (Care of the Dying Evaluation) post-death questionnaire was administered to family members who accompanied patients in their final days of life, assessing their perception of the quality of care provided to the patient and the level of support provided to the family.
- The CODETM questionnaire was a useful tool in evaluating the palliative care offered by hospitals to patients and can be applied to propose improvements in palliative care.

## CENTRAL MESSAGE

Patients with advanced cancer often experience a number of distressing symptoms and face psychological, social, and spiritual problems, in addition to physical symptoms. The end-of-life process is one of the most significant moments for individuals, their family members and caregivers, representing an emotional overload with several consequences. Palliative care (PC) represents an essential service to be implemented by health systems to support patients with irreversible diseases and without therapeutic possibilities, with a focus on symptom control and improvement of quality of life, preventing and relieving human suffering in several dimensions, including the final period of an end-stage illness, in a moment of progressive decline that is inevitably approaching death. This study compares the perception of caregivers of patients in palliative care offered by two public hospitals using the CODETM questionnaire.

## PERSPECTIVES

There is a need for an instrument that can constantly classify and qualify the care provided to oncological patients and their families in order to offer dignified, comprehensive and humanized care, as proposed by the CODETM questionnaire. There was no statistical difference between the caregivers’ perception of the care offered to patients between the two hospitals, being worse in the Emergency Unit.

## VISUAL ABSTRACT

**Figure:**
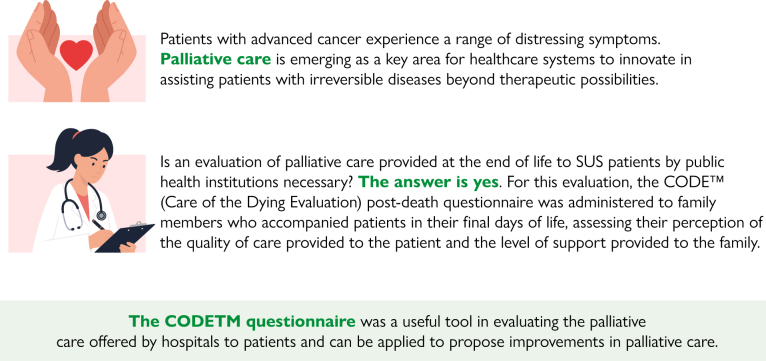

## RESUMO

**Racional:**

Pacientes com câncer avançado experimentam uma série de sintomas angustiantes. Os cuidados paliativos (CP) despontam como área essencial a ser implementada pelos sistemas de saúde, na assistência aos pacientes com doenças irreversíveis e fora de possibilidades terapêuticas.

**Objetivos:**

Comparar a percepção de cuidadores de pacientes em cuidados paliativos oferecidos por dois hospitais públicos por meio do questionário CODE^TM^; determinar a pontuação obtida pelo questionário e sua utilidade na avaliação dos cuidados paliativos oferecidos.

**Métodos:**

Foi aplicado o questionário pós-falecimento “Care of the Dying Evaluation” (CODE^TM^) aos familiares que acompanhavam os pacientes nos últimos dias, avaliando a percepção sobre a qualidade do atendimento prestado ao paciente e o nível de apoio à família.

**Resultados:**

Sem diferença estatística nos dados demográficos. Os participantes que receberam cuidados paliativos apresentaram maior escore na pontuação, bem como na unidade de enfermaria e UTI comparados à unidade de emergência. O valor de corte preditivo para a prática adequada de cuidados paliativos foi de 97 pontos, correspondendo a 78,6% da pontuação.

**Conclusões:**

Não houve diferença estatística entre a percepção dos cuidadores com os cuidados oferecidos aos pacientes entre os dois hospitais, sendo pior na unidade de Pronto Socorro. O valor de corte foi de 78,6% sendo considerado adequado e o questionário CODE^TM^ foi uma ferramenta útil na avaliação dos cuidados paliativos oferecidos pelos hospitais aos pacientes e pode ser aplicado para propor melhorias na atenção em cuidados paliativos. Portanto, há a necessidade de um instrumento que possa classificar e qualificar constantemente a assistência aos pacientes e seus familiares para ofertar uma atenção digna, integral e humanizada, como proposto pelo questionário CODE^TM^.

## INTRODUCTION

With the increase in life expectancy and population aging, the profile of diseases has changed considerably. Most of these diseases are related to the functional loss of physiology, resulting in loss of strength, vigor, and systemic reactions. Non-transmissible chronic diseases include cancer, which is among the main causes of death in Brazil and worldwide^5,8,14,30^.

Patients with advanced cancer often experience a number of distressing symptoms and face psychological, social, and spiritual problems, in addition to physical symptoms. The end-of-life process is one of the most significant moments for individuals, their family members, and caregivers, representing an emotional overload with several consequences^19^.

In this context, palliative care (PC) represents an essential service to be implemented by health systems to support patients with irreversible diseases and without therapeutic possibilities, with a focus on symptom control and improvement of quality of life, preventing and relieving human suffering in several dimensions, including the final period of an end-stage illness, in a moment of progressive decline that is inevitably approaching death^2,20^. This concept actually applies to patients and everyone around them who experience suffering together — family members, caregivers, and the health team, even if the concept and expectations of palliative care are different for each person/family^21,25,29^.

Thinking about palliative care should begin even before the treatment is instituted, as it can benefit the patient at the end of life; for example, resection surgery provides a better quality of life and survival for patients with incurable tumors^23^.

Another interesting fact is that the treatment of patients with tumors tends to have better results when carried out in specialized units and by multidisciplinary groups working concurrently^24^.

The creation of a palliative care team can impact hospital cuts, as demonstrated by the study of May et al., especially in the case of cancer patients, and can bring savings in the care of these patients^15^.

This study aimed to characterize the demographic data of patients in two hospitals using the CODE^TM^ questionnaire; identify, through the scores from each question and total questionnaire score, the presence of interaction between hospitals and care sectors; evaluate and compare scores of each question and total questionnaire score among participants who reported receiving or not receiving palliative care; determine a cutoff value for the score obtained in the questionnaire to rate hospital palliative care and then obtain a diagnosis of palliative care; and evaluate the usefulness of the questionnaire in the identification of palliative care.

This study aimed to compare the results between two public hospitals, using the CODE^TM^ questionnaire evaluating:

1. Palliative care offered to patients hospitalized in the ward, ICU, and emergency room, in the perception of the closest caregiver.
2. Determine a cutoff value for the score obtained in the questionnaire to rate hospital palliative care and then obtain a diagnosis of palliative care.
3. The usefulness of the questionnaire in identifying the palliative care offered to patients, in the perception of the closest caregiver.

## METHODS

The Fundação de Amparo à Pesquisa do Estado de São Paulo (FAPESP) and the Network of the European Union, Latin America, and Caribbean Countries on Joint Innovation and Research Activities (ERANet – LAC), a group that promotes joint activities that strengthen partnerships in science, technology, and innovation, signed a letter of commitment to conduct the ERANet-LAC 2015/16 Call, and a project was launched in the “health” area titled CODE™: quality of care for cancer patients as perceived by bereaved relatives, whose acronym is ERANet-LAC CODE™, aiming to improve and ensure the quality of life of end-stage cancer patients, offering care and support to patients and their families^15^.

This is an observational study that evaluates the perception of the care offered to the patient by bereaved family members during the postmortem mourning period, using quantitative data analysis and the evaluation method of the postmortem questionnaire “Care of the Dying Evaluation” (CODE^TM^), focusing on the last days of life and the period of immediate mourning. It seeks perceptions about the quality of patient care and the level of support for the family^10,16^.

The stage of translation of the CODE™ instrument from English into Portuguese was conducted using the protocol proposed by Kuliś et al.^12^, which included the initial translation, the back translation, and the review by experts, considering the participating country’s culture. The final version was the one considered in this study^22^.

The CODE^TM^ questionnaire has 42 questions divided into sections that include the evaluation of subareas, namely:

- Section A: Care received from nurses and physicians by the patient and their relatives/caregivers, such as issues related to personal hygiene and help with changes in positioning. In addition, this section evaluates privacy, cleanliness of the hospitalization environment, and the relationship with the team, taking into account the trust and time they made available to discuss the conditions of the patients with the family members.
- Section B: Pain and other symptom management takes into account the last 48 h of hospitalization, assessing the need for medication and team support for pain control, agitation, and respiratory distress.
- Section C: In communication with the health team, questions about participation in decisions about treatment and how to clarify the patient’s health conditions are evaluated.
- Section D: Evaluation of the emotional and spiritual support provided by the health team was evaluated by the degree of satisfaction with the support provided.
- Section E: The conditions of death section evaluates the support and assistance provided during and after the period of progression to death.
- Section F: The general impressions section also evaluates the support provided by the care team, taking into account the respect and dignity that has been dealt with in recent days.
- Section G: Personal information includes the degree of kinship, age, ethnic group, gender, and religious orientation of the patients and companions who answered the questionnaire. In addition, additional diagnostic information is also collected, in addition to cancer.

The score is divided among the sections, totaling 124 points, where the answers can vary according to the question in each section; that is, each question has a specific score according to the number of possible answers, with the minimum being zero and the maximum varying between 2, 3, 4, 5, and 6.

The list of patients diagnosed with cancer who died in the hospital was requested from the death service of Hospital Estadual Sumaré (HES) and the IT Service of Hospital de Clínicas da Unicamp (HCU). The patients were selected according to the criteria of inclusion, and the following information was collected: name, age, International Classification of Diseases (ICD), date of admission, date of death, and contact.

Inclusion criteria were family members or caregivers (participants) who accompanied the patient, participants aged over 18 years, participants interviewed 6–8 weeks after death, and participants able to answer the questionnaire for having experienced the last 2 days of the patient’s life.

Exclusion criteria were participant questionnaires with insufficient data, patient death before 48 h of admission, participant failure to answer the questionnaire, and participant discomfort during instrument application interrupting its completion.

Data collection was performed from October 2017 to June 2020, in person or by telephone, with only the last data collected during the COVID-19 pandemic period, which did not interfere with the total data collection.

The PC team at the HCU has one nurse and four medical professionals who are available to discuss the topic with the care team and the patient’s family members when requested via interconsultation by the medical team responsible for the care.

At the HES, the PC team is multidisciplinary, comprising a palliative care physician, a physical therapist, a speech therapist, a social worker, a psychologist, a nurse, and support from the hospital chaplain, and can be contacted by any member of the care team.

The researcher contacted the family member/caregiver during admission or later, 6–8 weeks after death, and then invited the contact to participate in the study and read and sign the informed consent form. After that, the interview and questionnaire completion were performed by telephone or in person.

The chi-square test was used in the analysis of characteristics of the study participants between the HES and the HCU. Two Generalized Linear Models (GLM) were used to analyze the scores of each question (Q1–Q32) and the total score obtained in the CODE™ questionnaire. The following independent variables were included in the model: hospital (HES and HCU) and sectors (general ward, ICU, and emergency room) in Model 1; and receipt of palliative care (yes and no) and sectors (general ward, ICU, and emergency room) in Model 2.

For both models, the Tweedie distribution was used for discrete dependent variables with positive values equal to or greater than zero, with the model estimated from identity link functions, and the binomial distribution was used for binary variables, with the model estimated from logit link functions.

Finally, discriminant validity of the CODE™ questionnaire was conducted to determine the total score, indicating adequate palliative care practice during the end-of-life process. The accuracy of the cutoff values was verified using a sensitivity test (true positive rate: correct identification of patients who reported receiving palliative care) and a specificity test (true negative rate: correct identification of patients who reported not receiving palliative care), construction of receiver operating characteristic curves, and analysis of the area under these curves (AUC) and their respective confidence intervals.

The accuracy of the discriminant value obtained was interpreted according to the AUC and classified as perfect (AUC=1), exceptional (0.9≤AUC<1), excellent (0.8≤AUC<0.9), acceptable (0.7≤AUC<0.8), and poor (AUC<0.7), considering that AUC is not statistically different from that obtained by chance for AUC values ≤0.5. To confirm the discriminant score, the Youden index was calculated, defined as the highest value observed for the following operation: sensitivity+specificity−131.32.

All analyses were performed using PASW Statistics 26.0 (SPSS Inc., Chicago, USA), adopting the significance level (α) of 5% (p<0.05).

This study was approved by the National Research Ethics Committee and the Research Ethics Committee of Unicamp under report n° 2.165.001, CAAE: 65309416.5.0000.5404, in accordance with Resolutions No. 466/2012 and 510/2016 of the National Health Council. Participation was voluntary, and all participants of the study read and signed the Free and Informed Consent Form in duplicate.

## RESULTS

**Figure 1.**
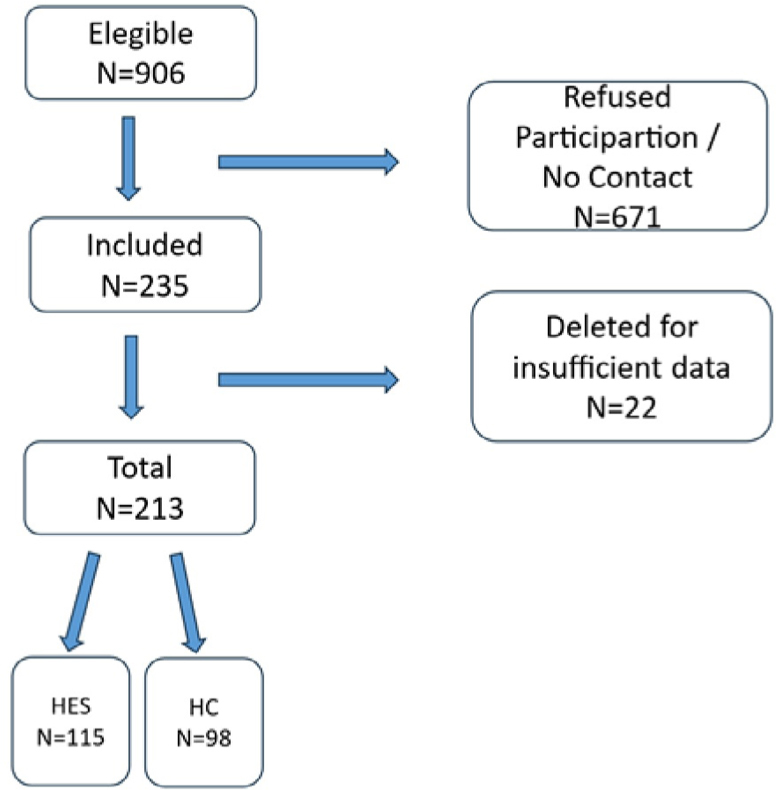
Total number of participants and the number of interviewees from each study institution.

HES: Hospital Estadual de Sumaré; HCU: Hospital de Clínicas da Universidade Estadual de Campinas.

During the study data collection period, there were 906 patient deaths in the two hospital institutions involving neoplastic diseases. Of these, 671 family members refused to respond to the questionnaire or were unable to be contacted. A total of 235 family members were eligible, with 22 of the data collected being incomplete and discarded from the case series, leaving 213 with complete data and eligible for analysis. Figure 1 shows eligible, excluded, and included patients, as well as the total number of participants and the number of interviewees from each study institution.

The number of interviews conducted by telephone (172, 73.19%) was significantly higher than in-person interviews (63, 26.81%).

Question 17 was not included in the analysis due to the high amount of missing data (90%), as well as question 26, which showed zero variance (all participants marked the same alternative).

Tables 1 and 2 show the demographic data of study participants and patients, showing no significant difference between the hospitals for any of the characterization variables (p≥0.05).

**Table 1:** Demographic data of the participants, presented as absolute and relative frequencies (%).

Participant information	HES		HCU	
	Number (%)		Number (%)	
Degree of kinship				
Husband/wife/partner	32	(27.8)	24	(24.5)
Son/daughter	47	(40.9)	39	(39.8)
Brother/sister	13	(11.3)	15	(15.3)
Son-in-law/daughter-in-law	11	(9.6)	6	(6.1)
Relative	2	(1.7)	5	(5.1)
Friend	0	(0.0)	3	(3.1)
Neighbor	0	(0.0)	0	(0.0)
Nursing team or home care	5	(4.3)	3	(3.1)
Private security	0	(0.0)	0	(0.0)
Other	5	(4.3)	3	(3.1)
Age (years)				
18–19	1	(0.9)	1	(1.0)
20–29	11	(9.6)	5	(5.1)
30–39	20	(17.4)	24	(24.5)
40–49	25	(21.7)	29	(29.6)
50–59	28	(24.3)	25	(25.5)
60–69	19	(16.5)	13	(13.3)
70–79	11	(9.6)	1	(1.0)
80–89	0	(0.0)	0	(0.0)
90 or more	0	(0.0)	0	(0.0)
Ethnicity				
White	84	(73.0)	55	(56.1)
Black	7	(6.1)	5	(5.1)
Yellow	0	(0.0)	1	(1.0)
Brown	24	(20.9)	36	(36.7)
Indigenous	0	(0.0)	0	(0.0)
No information	0	(0.0)	1	(1.0)
Sex				
Male	40	(34.8)	23	(23.5)
Female	75	(65.2)	75	(76.5)
Religious affiliation				
Catholic/Evangelical	105	(92.9)	84	(85.7)
Jewish	1	(0.9)	0	(0.0)
Jehovah’s Witness	0	(0.0)	0	(0.0)
Buddhist	0	(0.0)	0	(0.0)
Spiritist/Candomblé/Other religions	7	(6.2)	14	(14.3)

HES: Hospital Estadual de Sumaré; HCU: Hospital de Clínicas da Universidade Estadual de Campinas.

**Table 2:** Demographic data of the patients, presented as absolute and relative frequencies (%).

Patient information	HES		HCU	
	Number (%)		Number (%)	
Age (years)				
18–19	0	(0.0)	2	(2.0)
20–29	2	(1.7)	2	(2.0)
30–39	3	(2.6)	4	(4.1)
40–49	8	(7.0)	8	(8.2)
50–59	24	(20.9)	22	(22.4)
60–69	30	(26.1)	31	(31.6)
70–79	34	(29.6)	23	(23.5)
80–89	13	(11.3)	6	(6.1)
90 or more	1	(0.9)	0	(0.0)
Ethnicity				
White	87	(75.7)	65	(66.3)
Black	5	(4.3)	6	(6.1)
Yellow	1	(0.9)	1	(1.0)
Brown	22	(19.1)	26	(26.5)
Indigenous	0	(0.0)	0	(0.0)
No information	0	(0.0)	0	(0.0)
Sex				
Male	67	(58.3)	59	(60.2)
Female	48	(41.7)	38	(38.8)
No information	0	(0.0)	1	(1.0)
Religious affiliation				
Catholic/Evangelical	109	(94.8)	85	(85.9)
Jewish	1	(0.9)	1	(1.0)
Buddhist	0	(0.0)	0	(0.0)
Jehovah’s Witness	0	(0.0)	0	(0.0)
Spiritist/Candomblé/Other religions	5	(4.3)	13	(13.1)
Palliative care reported				
Yes	62	(53.9)	44	(44.4)
No	52	(45.2)	54	(54.5)
No information	1	(0.9)	1	(1.0)

HES: Hospital Estadual de Sumaré; HCU: Hospital de Clínicas da Universidade Estadual de Campinas.

Regarding the study participants, the most common degree of kinship in both hospitals was parent-child, representing 40.9% of HES participants and 39.8% of HUC participants. The most prevalent age group was 50–59 years. The predominant ethnicity and gender in both hospitals were white (73% HES; 56.1% HCU) and female (65.2% HES; 76.5% HCU). Participants were mostly of Catholic/Evangelical religion, with 92.5% at the HES and 85.7% at the HCU (Table 1).

About the study patients, the most prevalent age group was different at the HES (70–79 years) and the HCU (60–69 years), but no statistical difference was observed between them (p≥0.05). White (75.7% HES; 66.3% HCU) and male (58.3% HES; 60.2% HCU) patients were predominant in the sample, regardless of the study hospital. Patients were mostly of Catholic/Evangelical religion (94.8% HES; 85.9% HCU) (Table 2).

Care from the palliative care team at the HES occurred in 53.9% of cases, while at the HCU in 44.4% of cases, with no statistical significance between them, as indicated in Table 2.

### Comparisons between study hospitals and admission sectors

The analysis using the GLM showed a significant hospital*sector interaction only for Q24 (p=0.029), in section E, which addresses the death conditions of patients. Lower scores were reported at the HCU when compared to the HES in the ward sector (p=0.001) and in the ICU when compared to the ward of the HES (p=0.007) (Table 3).

**Table 3:** Comparisons of the scores in each question and the total score of the CODE questionnaire between hospitals and hospitalization sectors.

Issues (Q)	HES						HCU				
	Infirmary (n=60)		ICU (n=23)		Emergency (n=32)		Infirmary (n=81)		ICU (n=17)		Emergency (n=0)
	Mean	SD	Mean	SD	Mean	SD	Mean	SD	Mean	SD	
Q1	3.3	0.7*	3.5	0.5*	2.8	1.0	3.8	0.5‡	3.6	0.8‡	---------
Q2	3.3	0.7*	3.5	0.5*	2.8	1.0	3.8	0.4‡	3.6	0.8‡	---------
Q3	3.3	0.7*	3.5	0.5*	2.7	1.0	3.7	0.5‡	3.9	0.2‡	---------
Q4	3.2	0.8*	3.4	0.7*	2.5	1.1	3.5	1.0	3.4	1.0	---------
Q5	3.3	1.1	3.2	1.0	2.9	1.0	3.0	1.0	3.5	0.9	---------
Q6	3.4	0.9*	3.1	1.0*	2.6	0.9	3.3	1.0	3.4	1.2	---------
Q7	3.3	1.0*	3.1	1.0	2.8	1.0	3.5	0.9	3.4	0.9	---------
Q8	3.3	0.8*	3.4	0.6*	2.4	1.1	3.3	1.0	3.1	1.2	---------
Q9	3.3	0.8*	3.4	0.6*	2.6	1.1	3.6	0.8‡	3.8	0.4‡	---------
Q10	2.7	1.3	3.2	1.2	2.5	1.3	3.1	1.2	3.9	0.5	---------
Q11	3.3	1.0	3.6	0.8	3.2	1.0	3.7	0.8‡	4.0	0.0‡	---------
Q12	3.0	1.2	3.2	1.0	3.0	1.2	3.1	1.3	3.5	0.9	---------
Q13	3.3	1.0	3.6	0.8	3.4	0.9	3.6	0.9	3.6	1.1	---------
Q14	3.1	1.2	3.4	1.1	3.0	1.2	2.7	1.5	3.1	1.6	---------
Q15	3.4	1.0	3.9	0.4	3.1	1.1	3.5	1.0	3.5	1.1	---------
Q16	3.4	1.0	3.1	1.3	3.0	1.2	3.0	1.6	3.1	1.6	---------
Q17	---------		---------		---------		---------		---------		---------
Q18	2.1	2.0*	2.3	2.0	0.9	1.7	1.5	1.9‡	1.4	2.0‡	---------
Q19	3.3	0.5	3.2	0.7	2.9	0.8	3.1	0.5	3.1	0.7	---------
Q20	2.6	0.8	2.4	1.0	2.3	0.9	2.3	1.1	2.0	1.2	---------
Q21	3.2	0.6	3.2	1.0	2.8	1.0	3.2	1.0	3.0	1.3	---------
Q22	3.2	0.7	3.2	1.0	2.7	1.0	3.2	1.1	2.8	1.5	---------
Q23	3.3	1.6	3.0	1.8	2.8	1.9	3.2	1.6	2.8	1.9	---------
Q24	2.8	1.8	1.4	1.9†	1.9	2.0	1.7	2.0‡	1.9	2.1	---------
Q25	2.0	2.0	1.7	2.0	1.3	1.9	1.2	1.8	1.2	1.9	---------
Q26	---------		---------		---------		---------		---------		---------
Q27	3.3	1.4	2.9	1.6	2.4	1.9	3.9	0.6	3.6	1.1	---------
Q28	3.3	0.8	3.3	0.9	2.8	0.9	3.6	0.9	3.4	1.2	---------
Q29	3.9	0.7	3.8	0.8	3.5	1.3	3.8	0.9	3.3	1.6	---------
Q30.1	3.6	0.6*	3.3	0.9*	3.1	0.9	3.9	0.5‡	3.8	0.4‡	---------
Q30.2	3.5	0.7*	3.2	1.0*	2.8	1.1	3.8	0.6‡	3.6	0.8‡	---------
Q31	3.8	0.9	3.5	1.4	3.1	1.7	3.6	1.2	3.5	1.3	---------
Q32	3.6	0.5*	3.5	0.7	3.3	0.5	3.8	0.5	3.4	1.0	---------
Total (points)	99.2	15.5*	97.8	15.6*	83.7	20.6	99.9	12.9	99.3	14.0	---------
Total (%)	80.0	12.5*	78.9	12.6*	67.5	16.6	80.6	10.4	80.1	11.3	---------

HES: Hospital Estadual de Sumaré; HCU: Hospital de Clínicas da Universidade Estadual de Campinas; ICU: Intensive Care Unit; SD: standard deviation.

*Difference from the emergency (p<0.05)

†Difference from the infirmary (p<0.05)

‡Difference from the HES (p<0.05).

Regardless of the sector, at HCU, a higher score was obtained in section A, which evaluates the care received from nurses and physicians, for Q1 (p=0.034), Q2 (p=0.044), Q3 (p=0.002), and Q9 (p=0.035). In section B, which has questions about pain control and other symptoms, a statistical significance was found for Q11 (p=0.024), and in section F, about general impressions, for Q30.1 (p=0.007) and Q30.2 (p=0.034). In section C, which is data on health care communication, a lower score was observed for the HCU when compared to the HES for Q18 (p=0.038) (Table 3).

Table 3 shows that, regardless of the hospital, higher scores were observed for the ward and ICU compared to the emergency room in section A for Q1–Q4 (p<0.05 for all), Q6 (p<0.001 and p=0.015), Q8 (p<0.001 and p=0.002), Q9 (p<0.001 for both), and in section F for Q30.1 (p<0.001 and p=0.05), Q30.2 (p<0.001 and p=0.002), and total score (p<0.001 for both), as well as a higher score for the ward when compared to the emergency room for Q7 (p=0.002) in section A, Q18 (p=0.008) in section C, and Q32 (p=0.01) in section F.

### Comparisons between palliative care and hospitalization sectors

No significant interactions were observed between the palliative care sector (p≥0.05 for all). However, when comparing the scores between patients who received palliative care or not, regardless of the sector, statistical significance was observed in section A for Q1–Q7 (p<0.01 for all) and in section B for Q13 (p=0.042), according to Table 4.

**Table 4:** Comparisons of the scores in each question and total score of the CODE questionnaire among participants who reported having received or not received palliative care by hospitalization sectors.

Issues (Q)	Received palliative care								Did not receive palliative care							
	Infirmary (n=70)			ICU (n=25)			Emergency (n=11)		Infirmary (n=71)			ICU (n=15)			Emergency (n=20)	
Mean	SD		Mean	SD		Mean	SD	Mean	SD		Mean		SD	Mean	SD
Q1	3.7	0.6*		3.7	0.5*		3.4	0.9	3.5	0.6*,†		3.3		0.8*,†	2.6	0.9†
Q2	3.7	0.7*		3.7	0.5*		3.4	0.9	3.5	0.6*,†		3.4		0.8*,†	2.6	1.0†
Q3	3.7	0.6*		3.7	0.5*		3.3	0.9	3.4	0.6*,†		3.6		0.5*,†	2.4	1.0†
Q4	3.6	0.8*		3.4	0.9*		3.2	0.9	3.2	0.9*,†		3.3		0.8*,†	2.1	1.1†
Q5	3.4	1.1		3.6	0.8		3.1	1.0	2.9	1.0†		2.9		1.0†	2.8	1.0†
Q6	3.5	0.9*		3.5	0.9		3.1	1.0	3.2	1.0*,†		2.8		1.3†	2.3	0.7†
Q7	3.6	0.8*		3.4	1.0		3.3	1.0	3.2	1.0*,†		3.1		1.0†	2.5	0.9†
Q8	3.4	1.0*		3.3	0.8*		2.5	1.3	3.2	0.9*		3.3		1.0*	2.4	1.0
Q9	3.7	0.7*		3.6	0.5*		2.9	1.3	3.3	0.9*		3.5		0.6*	2.5	1.0
Q10	3.0	1.4		3.5	1.0		3.1	1.0	2.9	1.2		3.5		0.9	2.2	1.4
Q11	3.5	1.0		3.8	0.6		3.6	0.8	3.5	0.8		3.6		0.8	3.0	1.0
Q12	3.2	1.1		3.6	0.8		3.1	1.0	2.8	1.3		2.9		1.0	3.0	1.4
Q13	3.6	0.8		3.8	0.7		3.8	0.6	3.3	1.1†		3.3		1.2†	3.2	1.0†
Q14	3.1	1.1		3.4	1.1		2.9	1.0	2.7	1.5		3.1		1.7	3.0	1.4
Q15	3.4	1.0		3.8	0.6		3.1	1.0	3.5	1.0		3.6		1.1	3.1	1.2
Q16	3.3	1.2		3.4	1.3		3.5	1.3	3.0	1.5		2.7		1.6	2.7	1.2
Q17	---------			---------			---------		---------			---------			---------	
Q18	2.1		2.0	1.9		2.0	1.5	2.0	1.5		1.9	1.9	2.1		0.6	1.5
Q19	3.2		0.6	3.3		0.7	3.0	1.2	3.1		0.5	2.9	0.7		2.9	0.4
Q20	2.5		0.9	2.6		1.0	2.7	0.9	2.2		1.0†	1.7	1.1†		2.1	0.8†
Q21	3.3		0.9	3.3		1.1	3.2	1.0	3.1		0.9†	2.8	1.2†		2.7	1.0†
Q22	3.3		0.9	3.3		1.1	2.9	1.0	3.0		0.9†	2.6	1.5†		2.7	1.0†
Q23	3.5		1.3	3.5		1.3	3.6	1.2	2.9		1.8†	1.9	2.1†		2.4	2.0†
Q24	2.5		2.0	2.2		2.0	3.3	1.6	1.9		2.0†	0.5	1.4†		1.2	1.9†
Q25	1.8		2.0	1.6		2.0	2.2	2.1	1.2		1.9†	1.3	2.0†		0.8	1.6†
Q26	---------			---------			---------		---------			---------			---------	
Q27	3.7	1.0*		3.4	1.4		2.9	1.9	3.6		1.1*	2.8		1.5	2.2	1.9
Q28	3.6	0.8		3.5	0.9		3.1	0.8	3.3		0.9†	2.9		1.2†	2.7	1.0†
Q29	3.8	0.8		3.8	0.8		3.3	1.6	3.8		0.8	3.2		1.7	3.8	0.9
Q30.1	3.8	0.6*		3.6	0.5		3.4	0.9	3.7		0.6*,†	3.2			2.9	0.9†
Q30.2	3.7	0.7*		3.6	0.7		2.8	1.3	3.6		0.6*	3.0		1.1	2.8	1.0
Q31	3.8	0.9		3.8	0.8		3.3	1.6	3.6		1.2	2.9		1.8	3.2	1.6
Q32	3.8	0.4*		3.6	0.6		3.5	0.5	3.5		0.6*,†	3.1		1.0†	3.2	0.4†
Total (points)	103.7	13.9*		104.4	11.3*		95.8	17.5	95.5		13.1*,†	88.5		14.8*,†	78.15	19.7†
Total (%)	83.7	11.2*		84.2	9.1*		77.3	14.1	77.0		10.6*,†	71.3		11.9*,†	63.0	15.9†

ICU: Intensive Care Unit; SD: standard deviation;

*Difference from Emergency (p<0.05)

†Difference in relation to the group that received palliative care (p<0.05).

Also, in section D, which addresses emotional and spiritual support provided by the health care team, significant main effects were also identified for Q20-Q22 (p<0.05 for all), and in section E for Q23-Q25 (p<0.05 for all) and Q28 (p=0.040). In section F, about general impressions, statistical significance was observed for Q30.1 (p=0.008), Q32 (p<0.001), and total score (p<0.001), as indicated in Table 4.

Regardless of the receipt of palliative care, higher scores were observed in the ward and ICU when compared to the emergency room in section A for Q1–Q4 (p<0.05 for all), Q8 (p<0.001 and p=0.007), Q9 (p<0.001 and p=0.001), and total score (p<0.001 for both); as well as a higher score in the ward when compared to the emergency room in section A for Q6 (p=0.007) and Q7 (p=0.028), in section E for Q27 (p=0.035), and in section F for Q30.1 (p<0.001), Q30.2 (p<0.001), and Q32 (p=0.042) (Table 4).

### Validation of the CODE^TM^ questionnaire for the discrimination of the palliative care practice

The discriminant cutoff value of adequate palliative care practice, based on the total score of the CODE™ questionnaire, was significant (p<0.001) and rated as acceptable (AUC=0.73) when analyzing the entire sample. Then, a cutoff value of 78.6% of the total score (97 points) was obtained, whose equal or higher values indicate adequate palliative care practice, with 71.7% sensitivity and 63.2% specificity.

When the analysis was replicated in fractions of the total sample, represented by the two hospitals (HES and HCU), the discriminative power was maintained with minimal variations (AUC between 0.72 and 0.75), demonstrating stability of the cutoff value identified in the general sample for the identification of adequate palliative care practices using the CODE™ questionnaire in samples from different places (Table 5).

**Table 5:** Accuracy of the total score obtained in the CODE questionnaire for the identification of hospital palliative care practice.

Age group	AUC	95%CI	p-value	Cut*	Sensitivity (%)	Specificity (%)	Palliative care	
							Yes	No
All	0.73	0.66–0.79	<0.001	78.6	71.7	63.2	106	106
HES	0.75	0.65–0.84	<0.001	75.4	75.8	65.4	62	52
HCU	0.72	0.62–0.82	<0.001	78.6	84.1	53.7	44	54

AUC: Area under the characteristic operating curve of the receiver; 95%CI: 95% confidence interval; HES: Hospital Estadual de Sumaré; HCU: Hospital de Clinicas da Universidade Estadual de Campinas.

*Total score value (%) in the questionnaire CODE above which good palliative care practices are attributed.

## DISCUSSION

Learning how to handle a loss due to a chronic illness such as cancer is an issue that few people are willing to discuss and confront. Helping individuals with advanced and potentially fatal illnesses with their families in one of the most important moments of their lives is a health care model that has become increasingly challenging^1,18^.

The estimated incidence of cancer in Brazil is large; it is estimated that, in the 3-year period 2023–2025, the incidence will be 704 thousand new cases. Excluding non-melanoma skin cancer, 483,000 new cases will occur^26^.

In a study to evaluate the indication of palliative care in the elderly hospitalized in the Intensive Care Unit, it was observed that, of 594 medical records analyzed, cardiovascular diseases corresponded to 26.8%, followed by neoplasms at 20.2% and renal failure at 16.8% among the elderly hospitalized in the ICU, demonstrating the importance of the subject today^13^.

Palliative care is recognized as an approach that improves the quality of life of patients with end-stage illness and their families^18^. Physical, emotional, spiritual, and social distress must be assessed and controlled for better treatment and relief^28^. This support should be offered to patients with end-stage illness (from diagnosis to death) and their families (during the disease and in bereavement programs), as assessed in the CODE™ questionnaire^16^.

The number of interviews conducted by telephone was significantly higher than in-person interviews, which can be explained by the fact that family members were not able to return to the hospital, since many of them lived in other cities or states, especially in the last months of data collection from March 2020 to June 2020, due to the COVID-19 pandemic.

At the HCU, 44.9% of patients received support from the PC team, and at HES, PC was provided in 53.9% of cases, probably using a different team model between them.

Santos-Moura et al.^27^ highlight palliative care as a complex approach that aims to fulfill all dimensions of the patient and family members and the importance of a multidisciplinary team with a nurse, a psychologist, a physician, a social worker, a pharmacist, a nutritionist, a physical therapist, a speech therapist, an occupational therapist, a dentist, and a spiritual assistant. They observe that, in order to provide quality care, it is essential to offer harmonic and convergent support to the patient, which does not aim to achieve healing but rather an option of treatment and adequate care for these patients and their families^27^.

The epidemiological data of our investigation showed a mean age of 69.8 years for male white patients, in agreement with the study conducted by Francisco et al.^7^.

Regarding religion, more than 85% of Christian participants and patients were observed in both hospitals. In a meta-analysis study of randomized clinical trials, Xing et al.^31^ observed that spirituality, in addition to interfering with quality of life, helps reduce depression, hopelessness, and anxiety generated by cancer, in agreement with the study conducted by Gull and Kaur^9^, which demonstrated prayer as an efficient strategy to reduce anxiety in cancer patients.

In the comparison between hospitals and hospitalization sectors, interaction was observed for question 24, which addresses the explanation for the patient companion about what symptoms to expect when the patient is at the end of life. A lower score was observed at the HCU in the ward sector when compared to the HES, which can be explained by the multidisciplinary characteristic of the team and their accessibility.

At the HES, lower scores were also observed for Q24 in the ICU when compared to the ward, and this fact can be considered due to the profile of the hospitalization unit.

In our study, no significant interactions were observed between palliative care sectors, but when comparing the scores between patients who received palliative care or not, regardless of the sector, statistical significance was observed in some questions in sections A, B, D, E, and F that evaluated care received from nurses and physicians, control of pain and other symptoms, emotional and spiritual support offered during hospitalization, the patient’s death conditions, and general impressions of care of both hospitals.

These patients also presented a higher total score in the questionnaire when compared to those who did not receive care, highlighting the importance of providing comprehensive and humanized care to ensure quality of life in such an important stage for patients and their families.

Regardless of the receipt of palliative care, higher scores were observed in the ward and ICU when compared to the emergency room in section A and total score, as well as higher scores in the ward when compared to the emergency room in sections A, E, and F, a fact that was also justified by the profile of the hospitalization unit.

Medeiros et al.^17^ highlighted that the chaotic use of emergency care, crowded emergency services, and the lack of hospital beds can create several obstacles to quality care for both patients and the health team.

Then, we emphasize the importance of continuous assessment of care provided by the care team in each hospitalization unit — whether it is a ward, an ICU, or an emergency room. Also, an instrument is required that can constantly rate and qualify the care provided to patients and their families in order to offer dignified, comprehensive, and humanized care, as proposed by the CODE™ questionnaire.

The predictive cutoff value defined in this study to indicate adequate care practice was 97 points, corresponding to 78.6% of the total score. When the analysis was replicated in fractions of the total sample, represented by the two hospitals (HES and HCU), the discriminative power was maintained with minimal variations, demonstrating stability of the cutoff value identified in the general sample from different locations. The instrument proved to be safe for the evaluation, analysis, and discrimination of adequate provision of care to end-of-life patients and their families.

There is no cut-off score in the literature, nor in the validation of the questionnaire, that classifies the care provided, which was statistically elaborated and analyzed by this specific research.

Thus, a discriminative validation of the CODE^TM^ questionnaire was conducted to determine a total score indicative of the appropriate practice of palliative care. The accuracy of the cut-off values was verified through sensitivity tests (true positive rate: correct identification of patients who reported having received palliative care) and specificity (true negative rate: correct identification of patients who reported not having received palliative care); construction of characteristic curves of the receiver’s operation; and analysis of the area under these curves (AUC) and their respective confidence intervals. The accuracy of the discriminant value obtained was interpreted based on AUC and classified as: “perfect” (AUC=1), “exceptional” (0.9≤AUC<1), “excellent” (0.8≤AUC<0.9), “acceptable” (0.7≤AUC<0.8), and “poor” (AUC<0.7), taking into account that AUC is not statistically different from that obtained at random for AUC values≤0.5^11^. To confirm the discriminant score, the Youden index was calculated, defined as the highest value observed for the following operation: sensitivity+specificity−1^6,32^.

Discrimination of palliative care practice:

In the analysis of the identification of the discriminant cut-off value of the appropriate practice of palliative care, based on the total score of the CODE^TM^ questionnaire, the predictive power was significant (p<0.001) and classified as acceptable (AUC=0.73) when the entire sample was analyzed. Thus, a cut-off value corresponding to 78.6% of the total score (about 97 points) was obtained, whose values equal to or higher indicate adequate palliative care practice with a sensitivity of 71.7% and a specificity of 63.2%.

Considering this reality, Campos et al.^3^ reported that the palliative care team should be available to handle the pain experienced by family members while accompanying the patient during all the processes, which are focused on avoiding suffering at any cost, including, in most cases protecting, the patient from any information that may cause sadness and despair. The palliative care team itself, especially nursing, should receive support so that there is no marked wear and tear that can impact on their health and performance^4^.

In view of the above, the importance of proper care is identified, offering professionals, patients, and family members a dignified and comfortable environment to provide and receive end-of-life care, using instruments that can assess and rate the service quality, and always seeking to improve individualized and comprehensive care.

## CONCLUSIONS

1. This study did not demonstrate statistical significance observed among public hospitals when comparing the support of the palliative care team. The data showed that, regardless of the hospital and the receipt of palliative care, higher scores were observed in the ward and ICU when compared to the emergency room. The data also showed better scores, regardless of sector, for patients who received palliative care.
2. The discriminant cutoff value of adequate practice of palliative care, based on the total score of the CODE^TM^ questionnaire, corresponded to 78.6% of the total score.
3. The CODE^TM^ questionnaire proved to be an appropriate evaluation tool to improve the service provided, allowing the identification and resolution of failures and deficiencies in each institution, increasing hospital efficiency with the diagnosis and mapping of care processes in palliative care provided to patients in the two public institutions.

## Data Availability

The datasets generated and/or analyzed during the current study are available from the corresponding author upon reasonable request.
